# KRGDB: the large-scale variant database of 1722 Koreans based on whole genome sequencing

**DOI:** 10.1093/database/baz146

**Published:** 2020-03-04

**Authors:** Kwang Su Jung, Kyung-Won Hong, Hyun Youn Jo, Jongpill Choi, Hyo-Jeong Ban, Seong Beom Cho, Myungguen Chung

**Affiliations:** 1 Division of Biomedical Informatics, Center for Genome Science, National Institute of Health, KCDC, Cheongju 28159, Republic of Korea; 2 Healthcare R&D Division, Theragen Etex Bio Institute Co. LTD., Suwon 16229, Republic of Korea; 3 Thermo Fisher Scientific Solutions, Seoul 06349, Republic of Korea and; 4 Future Medicine Division, Korea Institute of Oriental Medicine, Daejeon 34054, Republic of Korea

## Abstract

Since 2012, the Center for Genome Science of the Korea National Institute of Health (KNIH) has been sequencing complete genomes of 1722 Korean individuals. As a result, more than 32 million variant sites have been identified, and a large proportion of the variant sites have been detected for the first time. In this article, we describe the Korean Reference Genome Database (KRGDB) and its genome browser. The current version of our database contains both single nucleotide and short insertion/deletion variants. The DNA samples were obtained from four different origins and sequenced in different sequencing depths (10× coverage of 63 individuals, 20× coverage of 194 individuals, combined 10× and 20× coverage of 135 individuals, 30× coverage of 230 individuals and 30× coverage of 1100 individuals). The major features of the KRGDB are that it contains information on the Korean genomic variant frequency, frequency difference between the Korean and other populations and the variant functional annotation (such as regulatory elements in ENCODE regions and coding variant functions) of the variant sites. Additionally, we performed the genome-wide association study (GWAS) between Korean genome variant sites for the 30×230 individuals and three major common diseases (diabetes, hypertension and metabolic syndrome). The association results are displayed on our browser. The KRGDB uses the MySQL database and Apache-Tomcat web server adopted with Java Server Page (JSP) and is freely available at http://coda.nih.go.kr/coda/KRGDB/index.jsp.

Availability: http://coda.nih.go.kr/coda/KRGDB/index.jsp

## Introduction

Advances in sequencing technology (next-generation sequencing [NGS]) permit rapid nucleotide sequencing of large sections of genomes to be achieved at a lower cost than using classical Sanger sequencing methodology (1). In 2012, using the NGS technique, the 1000 Genome Project (1000 Genomes) sequenced and presented the whole genome and exome sequence variants of 1092 individuals (2). Completion of this project led to the development of dramatically more efficient sequencing technologies and, ultimately, led to a stream of personal genome sequencing projects (3–10). As a part of the global stream, two Korean groups conducted the whole genome sequencing of a total of 11 individuals (11–12). However, the sample size was insufficient to establish and evaluate a comprehensive map of Korean common and rare variants, and thus it was difficult to use the genome variants for other genome studies.

Since 2009, the Center for Genome Science (CGS) of the Korea National Institute of Health (KNIH) has been reporting the findings of genome-wide association studies (GWASs), which have identified several epidemiological traits and diseases among Korean populations (13) and East Asian populations (14–15). The variants used in the GWASs were deposited in the Korean variant databases: KARE browser (16), Evo-SNP DB (17), and KGVDB (18). Given that the variant sites originated from comparison to the GRCh37 reference genome, which is not of Asian origin, studies on the Korean-specific variant sites were limited. Therefore, the CGS initiated the Korean Reference Genome project (KRG) in 2012 and has been conducting whole genome sequencing on a total of 1722 Korean individuals, wherein more than 32 million variants for the Korean population were identified, and a large proportion of the variants were detected for the first time. In this study, we constructed a database and web browser (the Korean Reference Genome Database [KRGDB]) for 27 million single nucleotide variants (SNVs) and 4.9 million short insertion/deletion variants (indels) in the first phase from 622 individuals (2012–2014). Additionally in the first phase, testing was performed in a genome-wide association study (GWAS) between Korean genome variant sites for the 30×230 individuals and three major common diseases (diabetes, hypertension and metabolic syndrome). The association results are displayed on our browser. Furthermore, 31 million SNVs and 4.2 million indels were identified in the second phase, from 1100 individuals (2015–2016). The KRGDB uses MySQL database and Apache-Tomcat web server adapted with Java Server Page (JSP) and is freely available at http://coda.nih.go.kr/coda/KRGDB/index.jsp.

## Materials and methods

### Sequencing subjects

In the first phase (2012–2014), 622 DNA samples of study subjects for the KRG were obtained from three different sources. The first source was the 63 participants of the Korea National Health and Nutrition Examination Survey (KNHANES), sequenced by 10× coverage depth. The second source was the 194 volunteers who participated in the Korean Genome Organization Conference, with 20× coverage. The third source was the 365 participants of a cohort study, known as the Ansan-Ansung cohort. The Ansan-Ansung cohort is a subset of the cohorts established by the Korean Genome Epidemiology Study (KoGES), in which 8842 individuals of the Ansan-Ansung cohort was previously genotyped by Affymetrix 5.0 SNP array and used in the GWASs (13). Of the 365 KoGES DNA samples, 135 individuals were sequenced by 10× coverage depth in 2012 and 20× coverage depth in 2013, and these were finally combined into 30× coverage depth (10×20×135). The remaining 230 KoGES DNA samples were sequenced by 30× coverage depth (30×230). The 30×230 participants approved the use of epidemiological and genotype data from the KoGES. In the second phase (2015–2016), 1100 individuals from the Korean Biobank Project were additionally sequenced and analyzed with 30× coverage. Table 1 summarizes the composition of the above KRG groups. HiSeq 2000 and HiSeq X Ten systems were used to produce DNA sequences in the first and second phases, respectively. Written informed consent was obtained from 1722 participants regarding the use of samples for whole genome sequencing, and this study was approved by the institutional review board of KNIH.

**Table 1:** The KRG individual groups

**Phase**	**No. of Individual**	**Description**	**Coverage**	**Platform**
The first phase (2012–2014)	63	Korea National Health and Nutrition Examination Survey	10×	HiSeq 2000
	194	Volunteers who participated in the Korean Genome Organization Conference	20×	
	230	The Ansan-Ansung cohort (epidemiological and genotype data)	30×	
	135	The Ansan-Ansung cohort (genotype) : merged 30× (10× in 2012 and 20× in 2013)	30× (10×+20×)	
The second phase (2015–2016)	1100	The Korean Biobank Project	30×	HiSeq X Ten

### Alignment and variant calling

The raw sequences were trimmed by Sickle-quality-based-trimming, a tool that uses sliding windows along with quality and length thresholds. Genome Reference Consortium Human Build 37 (GRCh37/hg19) was downloaded from the University of California Santa Cruz (UCSC) ftp server (ftp://hgdownload.cse.ucsc.edu/goldenPath/), and the sequencing reads produced by HiSeq™ 2000 and HiSeq™ X Ten sequencing systems were aligned to GRCh37 using Burrows-Wheeler Aligner (BWA) at default settings (19). We specified the quality threshold for read trimming using the –q 20 option to ensure high-quality reads for alignments. Thereafter, the BWA sample was used to generate alignments in the SAM format. PICARD was used for sorting, removing duplicate reads and converting from SAM to BAM format (http://picard.sourceforge.net/). In the first phase, SNVs and short indels were called using SAMtools ‘mpileup’ and ‘varFilter’ command with the –D 1000 option for specifying the coverage depth at min/max cutoffs of 3 and 1000, as well as options to disqualify SNPs that are too close to each other (20). The Isaac workflow was used to perform alignment and variant calling in the second phase (21).

### Variant annotation resources

To compare the allele frequency differences (AFD) between Korean and other populations, we used the HapMap III and 1000 Genome population alternative allele frequency, downloaded from the UCSC genome browser database (ftp://hgdownload.cse.ucsc.edu/). Simply, AFD is calculated by subtraction of the AF in KRG from those of other ethnic groups. Functional annotations were also conducted by ANNOVAR software (http://www.openbioinformatics.org/annovar/) (22). The genomic locations of the variants were annotated using the gene-based annotation implemented in ANNOVAR. The risk associated with the variants was predicted using the filter-based annotation implemented in ANNOVAR with non-synonymous variants (LJB version 2.3) for the effect on protein function (SIFT, PolyPhen, Muration Assesor) (23–25) and evolutional conservation (PhyloP, GERP, Siphy) (26–28).

### Analysis for the GWAS

The genomic variant risk associated with common diseases (diabetes, hypertension and metabolic syndrome) in the Korean population were analyzed using PLINK (version 1.07) (29). The association study was conducted by logistic regression with additive genetic model and covariates of age, sex and body mass index. The population characteristics are described in Supplementary Table 1.

## Results and discussion

### System architecture

**Figure 1:**
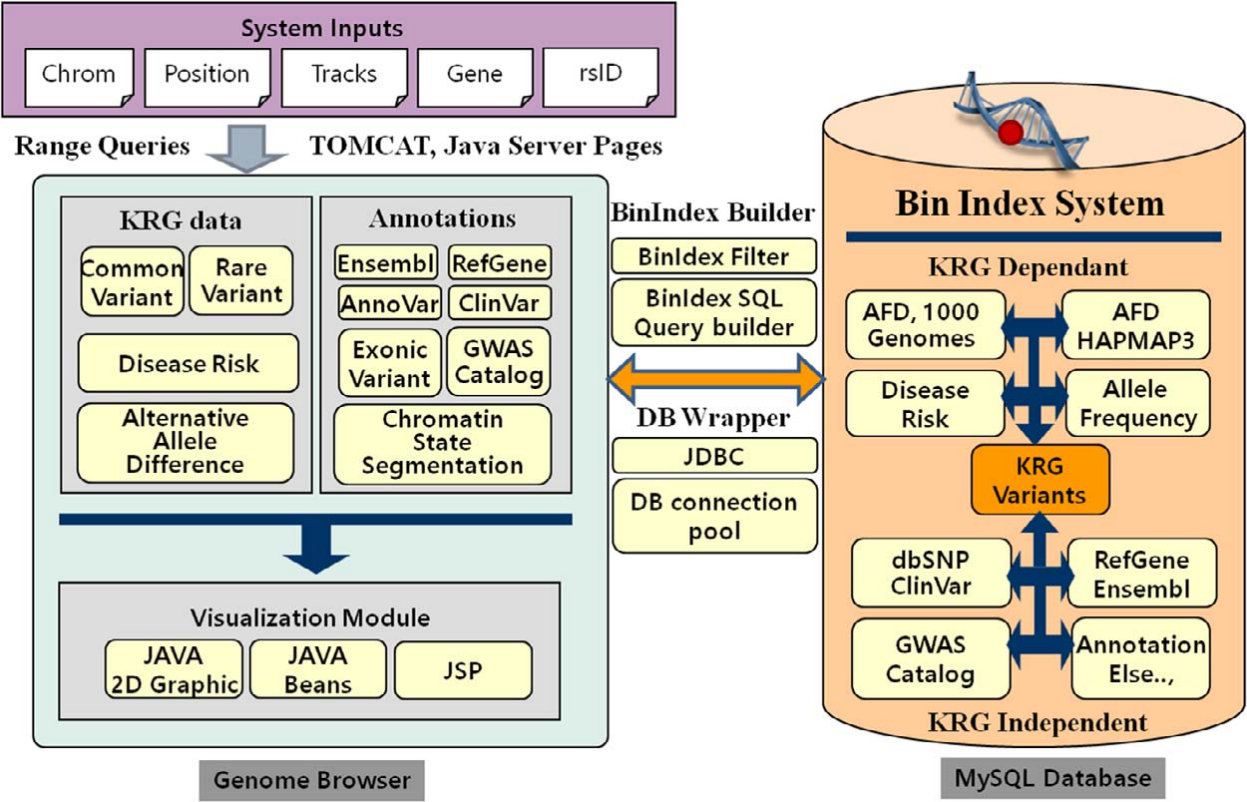
System architecture of KRGDB and Genome Browser. The system mainly consists of variation/annotation database and its genome browser.

The KRGDB is a web-based integrated variant resource for visualizing the 1722 Korean allele frequencies and related annotations simultaneously. The system is constructed with JSP and MySQL database on the Apache-Tomcat platform. The graphical charts showing variant regions were implemented using the pure Java2D graphic library and Java Beans technology. Users can easily browse variant regions and resources after inputting system requirements, such as chromosome number, absolute position of the chromosome, gene name, dbSNP rsID and optional tracks that users wish to study. Our genome browser provides not only variants of the first and second phase, but also variants of 30× coverage group (1465 individuals) and all-merged group (1722 individuals) from both phases (see Table 1). Figure 1 describes the system architecture of the KRGDB and genome browser. The system mainly consists of databases and its genome browser. The variation/annotation database and genome browser intuitively provide and display information in the database. The database includes SNVs that are derived from 1000 Genomes, International HapMap III project (30), dbSNP (31) and mainly KRG variants. The browser describes these variants databases and includes useful information for annotation, such as KRG disease allele risk, KRG exonic variants, gene information (RefGene, Ensembl) (32–33), ClinVar (34), GWAS Catalog (35) and chromatin state segmentation from the Broad Institute (36). The majority of source files have been downloaded from the UCSC genome annotation database (http://hgdownload.cse.ucsc.edu/goldenPath/hg19/database/), except for those associated with KRG disease allele risks (analyzed by PLINK software)(29) and exonic variants (analyzed by ANNOVAR software) (22). To accelerate browsing speed, the genome browser employs the Bin Indexing System (37), which is also used in the UCSC Genome browser. Detailed statistics and an explanation of current integrated resources will be discussed in the next section. The system has a focus on GRCh37, and the information in dbSNP build 151 is incorporated into the system. We, however, provide the lift-overed genomic positions of GRCh38 for convenient analysis between different assemblies. Certainly, our future research will contain remapping KRG individuals to GRCh38. The chain information file (from GRCh37 to GRCh38) has been downloaded from the UCSC liftOver web site (https://genome.ucsc.edu/cgi-bin/hgLiftOver). The information for annotation in KRG database will be approximately updated once every 3 months.

### Alternative allele frequency differences with other populations

**Figure 2:**
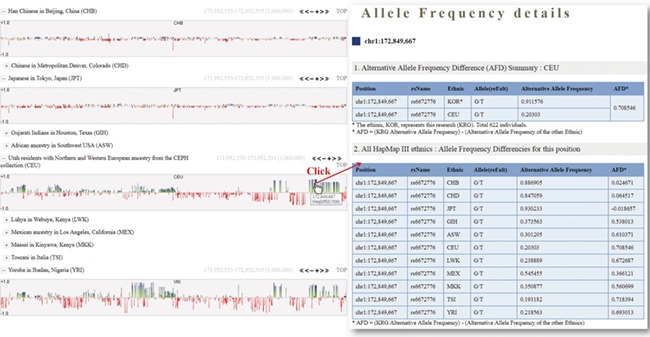
Alternative allele frequency difference between KRG and HapMap III ethnics. The horizontal axis denotes the genomic positions of the chosen chromosome (chr1).

**Figure 3:**
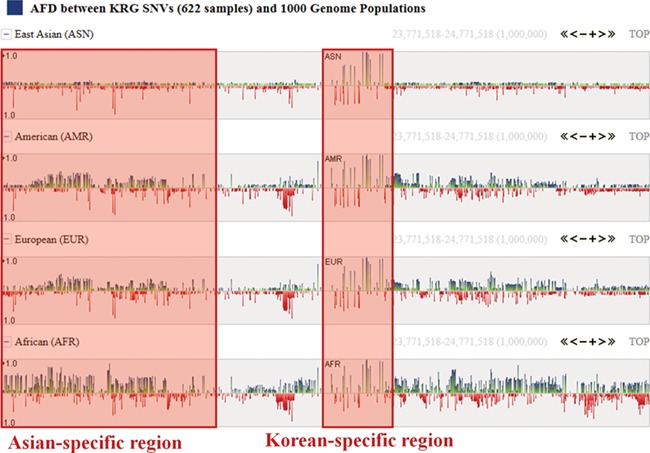
Alternative allele frequency difference between KRG and 1000 Genomes ethnics. The horizontal axis denotes the genomic positions of the chosen chromosome (chr1).

One of the useful functions of KRGDB is that the browser shows alternative allele frequency difference (AFD) for other ethnic groups. This is quite powerful because both identifying the ethnic-dependent allele frequency (AF) and showing distances among ethnic groups are possible with respect to genomic areas chosen by users. We therefore calculated AFDs from 622 individuals from the first phase. For example, Figure 2 shows AFD of four ethnic from HapMap III including Japanese (JPT), Chinese (CHB), CEPH European (CEU) and Yoruba African (YRI). Generally, Koreans are biologically much closer to JPT and CHB than CEU and YRI. AFDs, therefore, also reflect these aspects. In the charts, the green bars denote AFs more frequently found in the KRG studies, and the red bars denote AFs frequently found in other ethnic (non-Korean) groups. As another example, Figure 3 describes the AFD of four ethnic groups (Asian: ASN, Admixed American: AMR, European: EUR, African: AFR) from 1000 Genomes. The AFs of 1000 Genomes were downloaded from the ANNOVAR web site. We may predict that this is an Asian-specific variant region if the AFDs of the ASN in some regions are much smaller than those of other ethnic groups (AMR, EUR, AFR). Moreover, we would suspect a Korean-specific variant region if some area has large AFDs that are found in every ethnic group. However, it is clear that more validation and biological studies are required to finally establish this. Details of the AFD are shown in table form, with KRG’s AF and the AFDs of other ethnic groups, when users click on a specific red or green bar, in the same manner as described above.

### Associations of major common diseases

**Figure 4:**
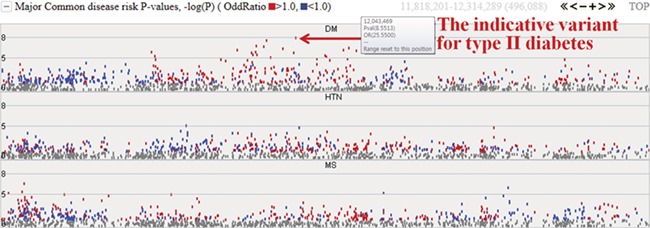
Disease risks of type II diabetes (DM), hypertension (HTN) and metabolic syndrome (MS). Each dot represents risk *P* values (–logP). The red and blue colour indicates odds ratio ≥1.0 and <1.0, respectively. The horizontal axis denotes the genomic positions of the chosen chromosome (chr1).

Among the 622 individuals from the first phase, we expanded our study to investigate the epidemiological and clinical data of 230 samples. The initial target diseases are type II diabetes, hypertension and metabolic syndrome. The system provides the −log*P* values for these three common diseases and will be extended to cover other diseases. Figure 4 represents risk *P* values (–log*P*) for major common diseases. The meaning of the –log*P* values are genome-wide significance levels (–log*P* ≥ 8) and suggestive levels (8 > –log*P* ≥ 5). Variants with higher −log*P* values may be indicative of each specific disease. Complex diseases are also simultaneously involved with other diseases. In chart form, it becomes clear that type II diabetes is deeply related to metabolic syndrome and hypertension. The colored dots represent the odds ratio for a specific variant, with red and blue dots indicating odds ratio ≥1.0 and <1.0, respectively.

### Additional information

We have provided useful annotation resources for use with the features described in the above sections; for example, chromatin state segmentation information from ENCODE/Broad, GWAS Catalog, ClinVar and ANNOVAR analysis for non-synonymous variants (LJB version 2.3). The graphical components in these charts intuitively show additional detailed information when the cursor of the mouse is clicked or hovers over the component; this applies also for other tracks. The addition of further supporting annotations is ongoing.

### Database statistics

The statistics of the variants database in Figure 1 are summarized in Supplementary Tables 2–6. In the first phase (2012–2014), KRG had a total 27 011 434 SNVs from 622 Koreans, including common and rare variants, and 31 750 003 SNVs from 1100 Koreans in the second phase (2015–2016). Common (alternative allele frequency ≥1%) variant distributions (8 672 646 SNVs from the first phase and 8 387 935 SNVs from the second phase) with respect to allele frequency range are described in Supplementary Table 2. In the first phase, the numbers of rare and short indel variants were 18 338 788 and 4 907 066, respectively. Likewise, 23 362 068 rare variants and 4 261 458 short indel variants were added in the second phase. We investigated 6 276 442 variants from 230 of 622 samples, to show disease association risk in the first phase. Supplementary Table 3 denotes the −log*P* distributions of the three diseases common among the Korean population: type II diabetes, hypertension and metabolic syndrome. Supplementary Tables 4 and 5 denote the number of variants in each range of alternative allele frequency difference (AFD). The results were listed for each ethnic group. The overlapping variants between KRG and 1000 Genomes (Supplementary Table 5, four ethnic groups) have more entries than HapMap III (Supplementary Table 4, 11 ethnic groups) because variants from the 1000 Genomes were generated by genome sequencing technology. Typically, the biologically farer ethnics from Korean have bigger absolute AFD values in Supplementary Tables 4 and 5.

## Conclusion

The main aim of the KRGDB is to provide a comprehensive map of Korean genetic variation to support studies of disease association and population genetics, and it has already been cited in analyses of variants found in individuals with genetic disorders (38–42). The KRGDB contains a large number of Korean variant sites, and a number of SNVs have not been included in the dbSNP151. Thus, our database offers the chance to confirm whether individual variants were already present in the general Korean population or whether they were present in a specific individual genome. Furthermore, the database, which includes alternative allele frequencies, can be used as a reference for understanding Korean or East Asian genomic diversity. Another expected application is the design of PCR primers and restriction enzyme sites. Moreover, the release of all summary statistics from the GWASs regarding three major common diseases (diabetes, hypertension and metabolic syndrome) is one of our useful outcomes. Users can perform meta-analysis using their own GWASs and our system. We believe that this database provides a quick reference that will aid the understanding of KRG features and facilitate research design based on KRG results.

## Supplementary Material

KRGDB_supplementary_v2_1_baz146

## References

[1] Shendure J. and Ji H. (2008) Next-generation DNA sequencing. *Nat. Biotechnol.*, 26, 1135–1145. 18846087 10.1038/nbt1486

[2] 1000 Genomes Project Consortium (2012) An integrated map of genetic variation from 1092 human genomes. *Nature*, 491, 56–65. 23128226 10.1038/nature11632 PMC3498066

[3] Wheeler D.A., Srinivasan M., Egholm M. et al. (2008) The complete genome of an individual by massively parallel DNA sequencing. *Nature*, 452, 872–876. 18421352 10.1038/nature06884

[4] Pushkarev D., Neff N.F. and Quake S.R. (2009) Single-molecule sequencing of an individual human genome. *Nat. Biotechnol.*, 27, 847–850. 19668243 10.1038/nbt.1561 PMC4117198

[5] Bentley D.R., Balasubramanian S., Swerdlow H.P. et al. (2008) Accurate whole human genome sequencing using reversible terminator chemistry. *Nature*, 456, 53–59. 18987734 10.1038/nature07517 PMC2581791

[6] Wang J., Wang W., Li R. et al. (2008) The diploid genome sequence of the Asian individual. *Nature*, 456, 60–65. 18987735 10.1038/nature07484 PMC2716080

[7] McKernan K.J., Peckham H.E., Costa G.L. et al. (2009) Sequence and structural variation in a human genome uncovered by short read, massively parallel ligation sequencing using two base encoding. *Genome Res.*, 19, 1527–1541. 19546169 10.1101/gr.091868.109 PMC2752135

[8] Fujimoto A., Nakagawa H., Hosono N. et al. (2010) Whole genome sequencing and comprehensive variant analysis of a Japanese individual using massively parallel sequencing. *Nat. Genet.*, 42, 931–936. 20972442 10.1038/ng.691

[9] Tong P., Prendergast J.G., Lohan A.J. et al. (2010) Sequencing and analysis of an Irish human genome. *Genome Biol.*, 11, R91. 20822512 10.1186/gb-2010-11-9-r91 PMC2965383

[10] Kitzman J.O., Mackenzie A.P., Adey A. et al. (2011) Haplotype-resolved genome sequencing of a Gujarati Indian individual. *Nat. Biotechnol*., 29, 59–63. 21170042 10.1038/nbt.1740 PMC3116788

[11] Ahn S.M., Kim T.H., Lee S. et al. (2009) The first Korean genome sequence and analysis: Full genome sequencing for a socio ethnic group. *Genome Res*., 19, 1622–1629. 19470904 10.1101/gr.092197.109 PMC2752128

[12] Kim J.I., Ju Y.S., Park H. et al. (2009) A highly annotated whole-genome sequence of a Korean individual. *Nature*, 460, 1011–1015. 19587683 10.1038/nature08211 PMC2860965

[13] Cho Y.S., Go M.J., Kim Y.J. et al. (2009) A large-scale genome-wide association study of Asian populations uncovers genetic factors influencing eight quantitative traits. *Nat. Genet*., 41, 527–534. 19396169 10.1038/ng.357

[14] Kim Y.J., Go M.J., Hu C. et al. (2011) Large-scale genome-wide association studies in East Asians identify new genetic loci influencing metabolic traits. *Nat. Genet.*, 43, 990–995. 21909109 10.1038/ng.939

[15] Cho Y.S., Chen C.H., Hu C. et al. (2012) Meta-analysis of genome-wide association studies identified eight new loci for type 2 diabetes in East Asians. *Nat. Genet*., 44, 67–72. 10.1038/ng.1019 PMC3582398 22158537

[16] Hong C.B., Kim Y.J., Moon S. et al. (2012) KAREBrowser: SNP database of Korea Association REsource project. *BMB Rep.*, 45, 47–50. 22281013 10.5483/bmbrep.2012.45.1.47

[17] Kim Y.U., Kim Y.J., Lee J.Y. and Park K. (2013) EvoSNP-DB: A database of genetic diversity in East Asian populations. *BMB Rep*., 46, 416–421. 23977990 10.5483/BMBRep.2013.46.8.191 PMC4133910

[18] Moon S., Jung K.S., Kim Y.J. et al. (2013) KGVDB: a population-based genomic map of CNVs tagged by SNPs in Koreans. *Bioinformatics*, 29, 1481–1483. 23626002 10.1093/bioinformatics/btt173 PMC3661059

[19] Li H. and Durbin R. (2010) Fast and accurate long-read alignment with Burrows-Wheeler transform. *Bioinformatics*, 26, 589–595. 20080505 10.1093/bioinformatics/btp698 PMC2828108

[20] Li H., Handsaker B., Wysoker A. et al. (2009) Genome Project Data Processing Subgroup: the sequence alignment/map format and SAMtools. *Bioinformatics*, 25, 2078–2079. 19505943 10.1093/bioinformatics/btp352 PMC2723002

[21] Raczy C., Petrovski R., Saunders C.T. et al. (2013) Isaac: ultra-fast whole-genome secondary analysis on Illumina sequencing platforms. *Bioinformatics*, 29(16), 2041–2043. 23736529 10.1093/bioinformatics/btt314

[22] Wang K., Li M. and Hakonarson H. (2010) ANNOVAR: functional annotation of genetic variants from high-throughput sequencing data. *Nucleic Acid Res.*, 38, e164. 20601685 10.1093/nar/gkq603 PMC2938201

[23] Kumar P., Henikoff S. and Ng P.C. (2009) Predicting the effects of coding non-synonymous variants on protein function using the SIFT algorithm. *Nat. Protoc.*, 4, 1073–1081. 19561590 10.1038/nprot.2009.86

[24] Adzhubei I.A., Schmidt S., Peshkin L. et al. (2010) A method and server for predicting damaging missense mutations. *Nat. Methods*., 7, 248–249. 20354512 10.1038/nmeth0410-248 PMC2855889

[25] Reva B., Antipin Y. and Sander C. (2011) Predicting the functional impact of protein mutations: application to cancer genomics. *Nucleic Acids Res*., 39, e118. 21727090 10.1093/nar/gkr407 PMC3177186

[26] Cooper G.M., Stone E.A., Asimenos G. et al. (2005) Distribution and intensity of constraint in mammalian genomic sequence. *Genome Res*., 15, 901–913. 15965027 10.1101/gr.3577405 PMC1172034

[27] Davydov E.V., Goode D.L., Sirota M. et al. (2010) Identifying a high fraction of the human genome to be under selective constraint using GERP++. *PloS Computational Biology*, 6, e1001025. 10.1371/journal.pcbi.1001025 PMC2996323 21152010

[28] Garber M., Guttman M., Clamp M. et al. (2009) Identifying novel constrained elements by exploiting biased substitution patterns. *Bioinformatics*, 25, i54–i62. 19478016 10.1093/bioinformatics/btp190 PMC2687944

[29] Purcell S., Neale B., Todd-Brown K. et al. (2007) PLINK: a tool set for whole-genome association and population-based linkage analyses. *Am. J. Hum. Genet*., 81, 559–575. 17701901 10.1086/519795 PMC1950838

[30] International HapMap III Consortium, Altshuler D.M., Gibbs R.A. et al. (2010) Integrating common and rare genetic variation in diverse human populations. *Nature*, 467, 52–58. 20811451 10.1038/nature09298 PMC3173859

[31] Sayers E.W., Barrett T., Benson D.A. et al. (2012) Database resources of the National Center for Biotechnology Information. *Nucleic Acids Res*., 40, D13–D25. 22140104 10.1093/nar/gkr1184 PMC3245031

[32] Pruitt K.D., Tatusova T., Klimke W. and Maglott D.R. (2009) NCBI Reference Sequences: current status, policy and new initiatives. *Nucleic Acids Res*., 37, D32–D36. 18927115 10.1093/nar/gkn721 PMC2686572

[33] Flicek P., Amode M.R., Barrell D. et al. (2014) Ensembl 2014. *Nucleic Acids Res*., 42, D749–D755. 24316576 10.1093/nar/gkt1196 PMC3964975

[34] Landrum M.J., Lee J.M., Riley G.R. et al. (2014) ClinVar: public archive of relationships among sequence variation and human phenotype. *Nucleic Acids Res*., 42, D980–D985. 24234437 10.1093/nar/gkt1113 PMC3965032

[35] Hindorff L.A., Sethupathy P., Junkins H.A. et al. (2009) Potential etiologic and functional implications of genome-wide association loci for human diseases and traits. *Proc. Natl. Acad. Sci. USA*, 106, 9362–9367. 19474294 10.1073/pnas.0903103106 PMC2687147

[36] Ernst J. and Kellis M. (2012) ChromHMM: automating chromatin-state discovery and characterization. *Nat. Methods*, 9, 215–216. 22373907 10.1038/nmeth.1906 PMC3577932

[37] Kent W.J., Sugnet C.W., Furey T.S. et al. (2002) The human genome browser at UCSC. *Genome Res*., 12, 996–1006. 12045153 10.1101/gr.229102 PMC186604

[38] Hosoda Y., Yoshikawa M., Miyake M. et al. (2018) CFH and VIPR2 as susceptibility loci in choroidal thickness and pachychoroid disease central serous chorioretinopathy. Proc. Natl. Acad. Sci. USA, 115(24), 6261–6266. 29844195 10.1073/pnas.1802212115 PMC6004488

[39] Lee C. G, Lee J., Lee M. et al. (2018) Multi-gene panel testing in Korean patients with common genetic generalized epilepsy syndromes. PLoS One, 13(6), e0199321. 10.1371/journal.pone.0199321 PMC6010271 29924869

[40] Kim B.J., Kim A.R., Lee C. et al. (2016) Discovery of CDH23 as a significant contributor to progressive postlingual sensorineural hearing loss in Koreans. PLoS One, 11(10), e0165680. 10.1371/journal.pone.0165680 PMC5085094 27792758

[41] Han K.H., Oh D.Y., Lee S. et al. (2017) ATP1A3 mutations can cause progressive auditory neuropathy: a new gene of auditory synaptopathy. Sci. Rep., 7, 16504. 10.1038/s41598-017-16676-9 PMC5705773 29184165

[42] Kim A. R, Chung J., Kim N. et al. (2017) The analysis of a frequent TMPRSS3 allele containing P.V116M and P.V291L in a cis configuration among deaf Koreans. Int. J. Mol. Sci., 18(11), 2246. 10.3390/ijms18112246 PMC5713216 29072634

