# KRGDB: the large-scale variant database of 1722 Koreans based on whole genome sequencing

**DOI:** 10.1093/database/baaa030

**Published:** 2020-04-29

**Authors:** Kwang Su Jung, Kyung-Won Hong, Hyun Youn Jo, Jongpill Choi, Hyo-Jeong Ban, Seong Beom Cho, Myungguen Chung

**Affiliations:** 1 Division of Biomedical Informatics, Center for Genome Science, National Institute of Health, KCDC, Cheongju 28159, Republic of Korea; 2 Healthcare R&D Division, Theragen Etex Bio Institute Co. LTD., Suwon 16229, Republic of Korea; 3 Thermo Fisher Scientific Solutions, Seoul 06349, Republic of Korea; 4 Future Medicine Division, Korea Institute of Oriental Medicine, Daejeon 34054, Republic of Korea

The original version of this article did not include Kyung-Won Hong's affiliation with Theragen Etex Bio Institute or Jongpill Choi's affiliation with Thermo Fisher Scientific Solutions, and incorrectly stated that Hyo-Jeong Ban was affiliated with both Theragen Etex and Thermo Fisher. These errors have now been corrected.

