# Supplementary material for: KRGDB: the large-scale variant database of 1722 Koreans based on whole genome sequencing
Source: Database (Oxford). 2020 Mar 4;2020:baz146. doi: 10.1093/database/baz146 (PMC7056612; doi:10.1093/database/baz146)
Supplement: KRGDB_supplementary_v2_1_baz146 [file krgdb_supplementary_v2_1_baz146.doc]

**Supplementary Tables**

**Supplementary Table 1**. 30×230 population characteristics

| Category | Variables | 30×230 (n =230) |
| --- | --- | --- |
| Sex | Male;Female | 128;102 |
| Age | Mean ± SD | 52.6±9.3 |
| Age group, year, n% | |  |
|  | 35-44 | 65 (28.3) |
|  | 45-54 | 66 (28.7) |
|  | 55-64 | 64 (27.8) |
|  | 65-74 | 35 (15.2) |
| Antropometric traits, mean±SD | |  |
|  | Weight (kg) | 63.5±11.7 |
|  | Height (cm) | 161.4±9.3 |
|  | Body mass index (kg/m2) | 24.3±3.3 |
|  | Waist circumference (cm) | 82.8±9.0 |
|  | Hip circumference (cm) | 93.6±6.6 |
|  | Waist-to-hip ratio | 0.88±0.07 |
|  | Systolic blood pressure (mmHg) | 113.8±16.1 |
|  | Distolic blood pressure (mmHg) | 74.3±10.9 |
| Biochemical traits, mean±SD | |  |
|  | Fasting glucose (㎎/㎗) | 88.8±23.3 |
|  | OGTT 2hr glucose (㎎/㎗) | 127.7±56.7 |
|  | Total cholesterol (㎎/㎗) | 198.5±38.6 |
|  | HDL cholesterol (㎎/㎗) | 45.7±10.1 |
|  | Triglycerides (㎎/㎗) | 163.4±93.3 |
| Common disease case | | Baseline |
| Type 2 diabetes, n(%) | |  |
|  | missing | 41 (17.8) |
|  | Case (GLU0≥126 and/or GLU120≥ 200) | 30 (13.0) |
|  | Control (GLU0 < 110 and GLU120 < 140) | 159 (69.1) |
| Hypertension, n(%) | |  |
|  | Missing | 18 (7.8) |
|  | Case (SBP ≥ 140 and/or DBP≥ 90) | 65 (28.3) |
|  | Control (SBP < 130 and DBP < 80) | 147 (63.9) |
| Metabolic syndrome, n(%)* | |  |
|  | missing | 0 (0.0) |
|  | Case | 58 (25.2) |
|  | Control | 172 (74.8) |

*Note.* Case was included the subjects with past history, treatments, drug therapy, and higher measurement value corresponding to each disease

*Metabolic syndrome defined by the presence of three or more of the following five components according the Adult Treatment Panel using waist circumference for Asians:

Central obesity (WC ≥ 90 cm for male or WC ≥ 80 cm for female)

Reduced HDL cholesterol (HDL < 40 ㎎/㎗ for male or HDL < 50 ㎎/㎗ for female)

Elevated Triglyceride level (TG ≥150 ㎎/㎗)

Elevated blood pressure (SBP ≥ 130 mmHg and/or DBP ≥ 85 mmHg)

Raised fasting glucose (GLU0 ≥ 100 ㎎/㎗)

**Supplementary Table 2.** Alternative Allele Frequency (AF) distribution of KRG Common Variants (alternative allele frequency ≥ 1%).

| (a) The First Phase (2012-2014) | (b) The Second Phase (2015-2016) |
| --- | --- |
| | AF range | # | % | | --- | --- | --- | | 1< ~ ≤5 | 2,291,950 | 26.4% | | 5< ~ ≤10 | 1,021,214 | 11.8% | | 10< ~ ≤15 | 691,513 | 8.0% | | 15< ~ ≤20 | 552,159 | 6.4% | | 20< ~ ≤25 | 465,368 | 5.4% | | 25< ~ ≤30 | 398,618 | 4.6% | | 30< ~ ≤35 | 347,359 | 4.0% | | 35< ~ ≤40 | 315,003 | 3.6% | | 40< ~ ≤45 | 288,552 | 3.3% | | 45< ~ ≤50 | 306,947 | 3.5% | | 50< ~ ≤55 | 230,802 | 2.7% | | 55< ~ ≤60 | 205,480 | 2.4% | | 60< ~ ≤65 | 186,033 | 2.1% | | 65< ~ ≤70 | 176,371 | 2.0% | | 70< ~ ≤75 | 170,285 | 2.0% | | 75< ~ ≤80 | 154,297 | 1.8% | | 80< ~ ≤85 | 142,691 | 1.6% | | 85< ~ ≤90 | 132,966 | 1.5% | | 90< ~ ≤95 | 132,912 | 1.5% | | 95< ~ ≤100 | 462,126 | 5.3% | | **SUM** | **8,672,646** | **100.0%** | | | AF range | # | % | | --- | --- | --- | | 1< ~ ≤5 | 2,234,658 | 26.7% | | 5< ~ ≤10 | 1,009,798 | 12.0% | | 10< ~ ≤15 | 694,976 | 8.3% | | 15< ~ ≤20 | 545,034 | 6.5% | | 20< ~ ≤25 | 452,019 | 5.4% | | 25< ~ ≤30 | 391,618 | 4.7% | | 30< ~ ≤35 | 337,593 | 4.0% | | 35< ~ ≤40 | 308,456 | 3.7% | | 40< ~ ≤45 | 278,397 | 3.3% | | 45< ~ ≤50 | 266,880 | 3.2% | | 50< ~ ≤55 | 218,547 | 2.6% | | 55< ~ ≤60 | 202,809 | 2.4% | | 60< ~ ≤65 | 182,506 | 2.2% | | 65< ~ ≤70 | 171,771 | 2.0% | | 70< ~ ≤75 | 161,353 | 1.9% | | 75< ~ ≤80 | 150,356 | 1.8% | | 80< ~ ≤85 | 139,667 | 1.7% | | 85< ~ ≤90 | 129,605 | 1.5% | | 90< ~ ≤95 | 135,353 | 1.6% | | 95< ~ ≤100 | 376,539 | 4.5% | | **SUM** | **8,387,935** | **100.0%** | |

**Supplementary Table 3.** Common disease risk. Genome-Wide significance level (–log*P* ≥ 8), Suggestive level ( 8 > –log*P* ≥ 5)

(A) Type II Diabetes

| **-log*P* range** | **#** | **%** |
| --- | --- | --- |
| ≤ 1.3 | 4,764,944 | 75.9 |
| 1.3< ~ ≤5.0 | 1,484,418 | 23.7 |
| 5.0< ~ ≤7.3 | 25,405 | 0.4 |
| > 7.3 | 1,675 | 0 |
| **Sum** | **6,276,442** | **100.00%** |

(B) Hypertension

| **-log*P* range** | **#** | **%** |
| --- | --- | --- |
| ≤ 1.3 | 4,614,630 | 73.5 |
| 1.3< ~ ≤5.0 | 1,635,134 | 26.1 |
| 5.0< ~ ≤7.3 | 24,827 | 0.4 |
| > 7.3 | 1,851 | 0 |
| **Sum** | **6,276,442** | **100.00%** |

(C) Metabolic Syndrome

| **-log*P* range** | **#** | **%** |
| --- | --- | --- |
| ≤ 1.3 | 4,705,217 | 75 |
| 1.3< ~ ≤5.0 | 1,546,434 | 24.6 |
| 5.0< ~ ≤7.3 | 23,316 | 0.4 |
| > 7.3 | 1,475 | 0 |
| **Sum** | **6,276,442** | **100.00%** |

**Supplementary Table 4.** KRG Alternative Allele Frequency Difference (AFD) with HAPMAP III 11 Populations.

| * JPT | | | * CHB | | |
| --- | --- | --- | --- | --- | --- |
| **AFD** | **#** | **%** | **AFD** | **#** | **%** |
| –100< ~ ≤–75 | 4,222 | 0.15 | –100< ~ ≤–75 | 4,222 | 0.15 |
| –75< ~ ≤–50 | 4,481 | 0.16 | –75< ~ ≤–50 | 4,489 | 0.16 |
| –50< ~ ≤–25 | 9,768 | 0.34 | –50< ~ ≤–25 | 9,588 | 0.33 |
| –25< ~ ≤0 | 1,506,264 | 52.57 | –25< ~ ≤0 | 1,528,264 | 53.32 |
| 0< ~ ≤25 | 1,328,371 | 46.36 | 0< ~ ≤25 | 1,306,982 | 45.6 |
| 25< ~ ≤50 | 8,638 | 0.3 | 25< ~ ≤50 | 8,652 | 0.3 |
| 50< ~ ≤75 | 2,134 | 0.07 | 50< ~ ≤75 | 2,133 | 0.07 |
| 75< ~ ≤100 | 1,635 | 0.06 | 75< ~ ≤100 | 1,633 | 0.06 |
| **Sum** | **2,865,513** | **100.00%** | **Sum** | **2,865,963** | **100.00%** |
| * CHD | | | * GIH | | |
| **AFD** | **#** | **%** | **AFD** | **#** | **%** |
| –100< ~ ≤–75 | 256 | 0.02 | –100< ~ ≤–75 | 233 | 0.02 |
| –75< ~ ≤–50 | 452 | 0.04 | –75< ~ ≤–50 | 3,429 | 0.26 |
| –50< ~ ≤–25 | 1,690 | 0.13 | –50< ~ ≤–25 | 78,970 | 6.03 |
| –25< ~ ≤0 | 686,395 | 53.95 | –25< ~ ≤0 | 571,152 | 43.58 |
| 0< ~ ≤25 | 583,205 | 45.84 | 0< ~ ≤25 | 558,485 | 42.61 |
| 25< ~ ≤50 | 154 | 0.01 | 25< ~ ≤50 | 93,706 | 7.15 |
| 50< ~ ≤75 | 16 | 0 | 50< ~ ≤75 | 4,617 | 0.35 |
| 75< ~ ≤100 | 10 | 0 | 75< ~ ≤100 | 28 | 0 |
| **Sum** | **1,272,178** | **100.00%** | **Sum** | **1,310,620** | **100.00%** |
| * ASW | | | * CEU | | |
| **AFD** | **#** | **%** | **AFD** | **#** | **%** |
| –100< ~ ≤–75 | 966 | 0.07 | –100< ~ ≤–75 | 4,716 | 0.17 |
| –75< ~ ≤–50 | 22,893 | 1.71 | –75< ~ ≤–50 | 24,574 | 0.87 |
| –50< ~ ≤–25 | 148,155 | 11.09 | –50< ~ ≤–25 | 224,776 | 7.91 |
| –25< ~ ≤0 | 479,170 | 35.86 | –25< ~ ≤0 | 1,095,603 | 38.57 |
| 0< ~ ≤25 | 492,800 | 36.88 | 0< ~ ≤25 | 1,176,470 | 41.42 |
| 25< ~ ≤50 | 163,760 | 12.26 | 25< ~ ≤50 | 280,104 | 9.86 |
| 50< ~ ≤75 | 27,367 | 2.05 | 50< ~ ≤75 | 31,415 | 1.11 |
| 75< ~ ≤100 | 989 | 0.07 | 75< ~ ≤100 | 2,610 | 0.09 |
| **Sum** | **1,336,100** | **100.00%** | **Sum** | **2,840,268** | **100.00%** |

- JPT : Japanese in Tokyo, Japan

- CHB : Han Chinese in Beijing, China

- CHD : Chinese in Metropolitan Denver, Colorado

- GIH : Gujarati Indians in Houston, Texas

- ASW : African ancestry in Southwest USA

- CEU : Utah residents with Northern and Western European ancestry from the CEPH collection

**Supplementary Table 4.** Continued.

| * LWK | | | * MEX | | |
| --- | --- | --- | --- | --- | --- |
| **AFD** | **#** | **%** | **AFD** | **#** | **%** |
| –100< ~ ≤–75 | 3,045 | 0.23 | –100< ~ ≤–75 | 198 | 0.02 |
| –75< ~ ≤–50 | 36,894 | 2.82 | –75< ~ ≤–50 | 2,811 | 0.22 |
| –50< ~ ≤–25 | 163,672 | 12.51 | –50< ~ ≤–25 | 70,312 | 5.56 |
| –25< ~ ≤0 | 435,886 | 33.32 | –25< ~ ≤0 | 551,553 | 43.59 |
| 0< ~ ≤25 | 454,294 | 34.73 | 0< ~ ≤25 | 555,989 | 43.94 |
| 25< ~ ≤50 | 172,165 | 13.16 | 25< ~ ≤50 | 81,133 | 6.41 |
| 50< ~ ≤75 | 39,251 | 3 | 50< ~ ≤75 | 3,230 | 0.26 |
| 75< ~ ≤100 | 2,962 | 0.23 | 75< ~ ≤100 | 28 | 0 |
| **Sum** | **1,308,169** | **100.00%** | **Sum** | **1,265,254** | **100.00%** |
| * MKK | | | * TSI | | |
| **AFD** | **#** | **%** | **AFD** | **#** | **%** |
| –100< ~ ≤–75 | 1,083 | 0.08 | –100< ~ ≤–75 | 442 | 0.03 |
| –75< ~ ≤–50 | 22,666 | 1.72 | –75< ~ ≤–50 | 10,674 | 0.82 |
| –50< ~ ≤–25 | 147,979 | 11.21 | –50< ~ ≤–25 | 114,674 | 8.8 |
| –25< ~ ≤0 | 476,153 | 36.06 | –25< ~ ≤0 | 506,537 | 38.88 |
| 0< ~ ≤25 | 485,293 | 36.75 | 0< ~ ≤25 | 518,406 | 39.79 |
| 25< ~ ≤50 | 159,592 | 12.09 | 25< ~ ≤50 | 136,712 | 10.49 |
| 50< ~ ≤75 | 26,567 | 2.01 | 50< ~ ≤75 | 14,787 | 1.14 |
| 75< ~ ≤100 | 1,048 | 0.08 | 75< ~ ≤100 | 512 | 0.04 |
| **Sum** | **1,320,381** | **100.00%** | **Sum** | **1,302,744** | **100.00%** |
| * YRI | | |  |  |  |
| **AFD** | **#** | **%** |  |  |  |
| –100< ~ ≤–75 | 11,799 | 0.42 |  |  |  |
| –75< ~ ≤–50 | 81,718 | 2.91 |  |  |  |
| –50< ~ ≤–25 | 326,095 | 11.59 |  |  |  |
| –25< ~ ≤0 | 878,367 | 31.23 |  |  |  |
| 0< ~ ≤25 | 1,051,942 | 37.4 |  |  |  |
| 25< ~ ≤50 | 366,210 | 13.02 |  |  |  |
| 50< ~ ≤75 | 87,490 | 3.11 |  |  |  |
| 75< ~ ≤100 | 9,257 | 0.33 |  |  |  |
| **Sum** | **2,812,878** | **100.00%** |  |  |  |

- LWK : Luhya in Webuye, Kenya

- MEX : Mexican ancestry in Los Angeles, California

- MKK : Maasai in Kinyawa, Kenya

- TSI : Toscani in Italia

- YRI : Yoruba in Ibadan, Nigeria

**Supplementary Table 5.** KRG Alternative Allele Frequency Difference (AFD) with 1000 Genome 4 Populations.

| * ASN | | | * AMR | | |
| --- | --- | --- | --- | --- | --- |
| **AFD** | **#** | **%** | **AFD** | **#** | **%** |
| –100< ~ ≤–75 | 6,578 | 0.06 | –100< ~ ≤–75 | 5,034 | 0.06 |
| –75< ~ ≤–50 | 15,634 | 0.15 | –75< ~ ≤–50 | 24,784 | 0.31 |
| –50< ~ ≤–25 | 52,031 | 0.5 | –50< ~ ≤–25 | 337,089 | 4.26 |
| –25< ~ ≤0 | 6,250,034 | 59.86 | –25< ~ ≤0 | 3,759,553 | 47.53 |
| 0< ~ ≤25 | 4,109,715 | 39.36 | 0< ~ ≤25 | 3,372,738 | 42.64 |
| 25< ~ ≤50 | 5,521 | 0.05 | 25< ~ ≤50 | 392,986 | 4.97 |
| 50< ~ ≤75 | 1,030 | 0.01 | 50< ~ ≤75 | 16,751 | 0.21 |
| 75< ~ ≤100 | 275 | 0 | 75< ~ ≤100 | 425 | 0.01 |
| **Sum** | **10,440,818** | **100.00%** | **Sum** | **7,909,360** | **100.00%** |
| * EUR | | | * AFR | | |
| **AFD** | **#** | **%** | **AFD** | **#** | **%** |
| –100< ~ ≤–75 | 5,628 | 0.07 | –100< ~ ≤–75 | 11,640 | 0.15 |
| –75< ~ ≤–50 | 49,571 | 0.64 | –75< ~ ≤–50 | 137,693 | 1.74 |
| –50< ~ ≤–25 | 488,654 | 6.31 | –50< ~ ≤–25 | 714,779 | 9.03 |
| –25< ~ ≤0 | 3,326,167 | 42.98 | –25< ~ ≤0 | 3,214,541 | 40.63 |
| 0< ~ ≤25 | 3,234,109 | 41.79 | 0< ~ ≤25 | 2,918,626 | 36.89 |
| 25< ~ ≤50 | 577,967 | 7.47 | 25< ~ ≤50 | 757,285 | 9.57 |
| 50< ~ ≤75 | 55,594 | 0.72 | 50< ~ ≤75 | 147,987 | 1.87 |
| 75< ~ ≤100 | 1,578 | 0.02 | 75< ~ ≤100 | 9,122 | 0.12 |
| **Sum** | **7,739,268** | **100.00%** | **Sum** | **7,911,673** | **100.00%** |

* Asian: ASN, Admixed American: AMR, European: EUR, African: AFR
